# Selecting effective siRNA sequences by using radial basis function network and decision tree learning

**DOI:** 10.1186/1471-2105-7-S5-S22

**Published:** 2006-12-18

**Authors:** Shigeru Takasaki, Yoshihiro Kawamura, Akihiko Konagaya

**Affiliations:** 1 RIKEN Genomic Sciences Center (GSC), Suehiro-cho 1-7-22-E216, Tsurumi-ku, Yokohama, Kanagawa, 230-0045, Japan

## Abstract

**Background:**

Although short interfering RNA (siRNA) has been widely used for studying gene functions in mammalian cells, its gene silencing efficacy varies markedly and there are only a few consistencies among the recently reported design rules/guidelines for selecting siRNA sequences effective for mammalian genes. Another shortcoming of the previously reported methods is that they cannot estimate the probability that a candidate sequence will silence the target gene.

**Results:**

We propose two prediction methods for selecting effective siRNA target sequences from many possible candidate sequences, one based on the supervised learning of a radial basis function (RBF) network and other based on decision tree learning. They are quite different from the previous score-based siRNA design techniques and can predict the probability that a candidate siRNA sequence will be effective. The proposed methods were evaluated by applying them to recently reported effective and ineffective siRNA sequences for various genes (15 genes, 196 siRNA sequences). We also propose the combined prediction method of the RBF network and decision tree learning. As the average prediction probabilities of gene silencing for the effective and ineffective siRNA sequences of the reported genes by the proposed three methods were respectively 65% and 32%, 56.6% and 38.1%, and 68.5% and 28.1%, the methods imply high estimation accuracy for selecting candidate siRNA sequences.

**Conclusion:**

New prediction methods were presented for selecting effective siRNA sequences. As the proposed methods indicated high estimation accuracy for selecting candidate siRNA sequences, they would be useful for many other genes.

## Background

Although RNA interference (RNAi) has been successfully used for studying gene functions in both plants and invertebrates, many practical obstacles need to be overcome before it becomes an established tool for use in mammalian systems [1-6]. One of the important problems is designing effective siRNA sequences for target genes. The short interfering RNA (siRNA) responsible for RNA interference varies markedly in its gene silencing efficacy in mammalian genes, where the gene silencing effectiveness depends very much on the target sequence positions (sites) selected from the target gene [7,8]. Since different siRNAs synthesized for various positions induce different levels of gene silencing, the selection of the target sequence is critical to the effectiveness of the siRNA. We therefore need useful criteria for gene silencing efficacy when we are designing siRNA sequences [9,10].

Zamore et al. and Jayasena et al. showed that 5' end of the antisense strand that was incorporated into RNA-induced silencing complex (RISC) more efficiently was less tightly paired to its complement and began with an A-T pair, whereas the strand incorporated less efficiently had a G-C terminus [11,12]. Other factors reported to be related to gene silencing efficacy are GC content, sequence features, specific motif sequences and secondary structures of mRNA. Several siRNA design rules/guidelines using efficacy-related factors have been reported [13-17].

Although sequence characteristics for siRNA designs seem to be the most important factor determining effective siRNA sequences, there are few consistencies among the reported rules/guidelines [18-22]. This implies that these rules/guidelines might result in the generation of many candidates and thus make it difficult to extract a few for synthesizing siRNAs. Furthermore, there is in RNAi a risk of off-target regulation: a possibility that the siRNA will silence other genes whose sequences are similar to that of the target gene. When we use gene silencing for studying gene functions, we have to first somehow select high-potential siRNA candidate sequences and then eliminate possible off-target ones [23].

Here we therefore focus on identifying high-potential siRNA sequences from many possible candidates and propose the prediction methods for selecting effective siRNA target sequences from many possible candidate sequences by using the radial basis function (RBF) technique and decision tree learning of a large number known effective and ineffective siRNAs [24-26]. We also propose the combined prediction method of the RBF network and decision tree learning. The effectiveness of the proposed methods were confirmed by using them to evaluate siRNA sequences recently reported to effectively or ineffectively suppress the expression of various genes (see Methods). As the average prediction probabilities of gene silencing for the effective and ineffective siRNA sequences of the reported genes by the proposed three methods were respectively 65% and 32%, 56.6% and 38.1%, and 68.5% and 28.1%, the methods imply high estimation accuracy for selecting candidate siRNA sequences. Although the proposed methods are different from the previous scoring methods and are therefore difficult to compare with them, the evaluation results indicate that the proposed methods would be useful for many other genes. They will therefore be useful for selecting siRNA sequences for mammalian genes.

## Results and Discussion

We propose two prediction methods for selecting effective siRNA sequences from many possible candidate sequences, one based on the supervised learning of RBF and other based on the learning of decision tree.

### Learning based on the RBF network and the decision tree

A radial basis function (RBF) network is a type of artificial network for application to problems of supervised learning, such as regression, classification and time series prediction. As RBF networks are nonparametric models, there is no *a priori *knowledge about the function that is to be used to fit the training set [24,25]. RBF networks are supervised learning models with a single middle layer of units. They are similar back propagation neural networks but usually faster to train because the RBFs used in the units mean that fewer weight adjustments are needed. Also, RBF networks tend to be more resistant to noisy data than back propagation networks. Decision tree learning is one of the most widely used and practical methods for inductive inference. A decision tree is a tree in which each branch node represents a choice between a number of alternatives, and each leaf node represents a classification or decision [26].

The proposed algorithms of the RBF network and the decision tree learning for selecting siRNA sequences effective are described in Methods.

### Verification of the proposed methods

**Figure 1:**
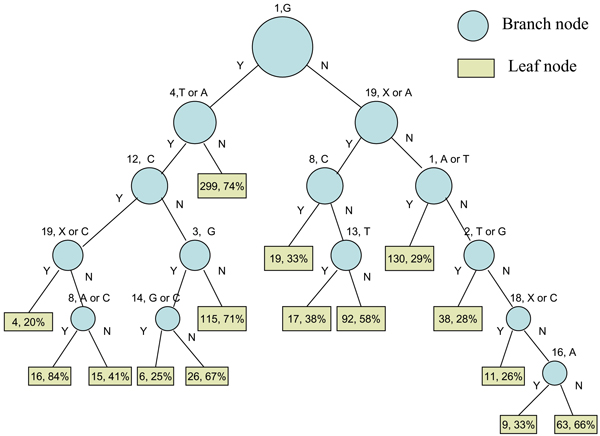
Decision tree diagram for known 860 effective and 860 ineffective siRNA sequences. The top of the branch node indicates the position and nucleotide attribute, e.g., "4, T or A" represents the cDNA position 4 with the nucleotide T or A. "X" indicates an arbitrary nucleotide, i.e., A, G, C or T. The bottom of the branch node shows yes (Y) and no (N). The leaf node indicates the number of effective siRNA sequences and its percentage, e.g., "299, 74%" means that the number of effective siRNA sequences is 299 and its percentage indicates 74% (= 299/404).

**Figure 2:**
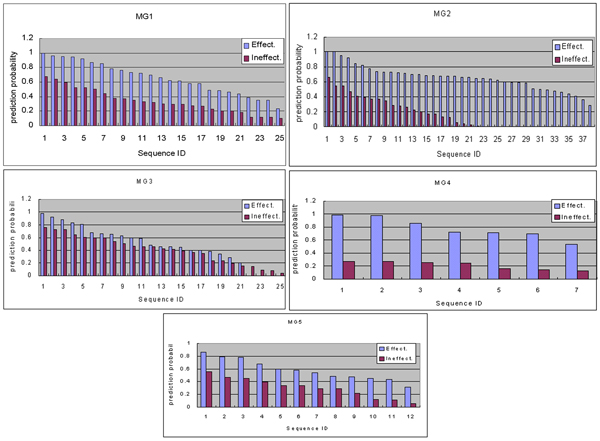
Prediction probability distributions of siRNA sequences effective and ineffective for MG1 to MG5 by the proposed RBF method. The probabilities for effective ("Effect") and ineffective("Ineffect") siRNAs computed by using the proposed RBF method are shown for (a) MG1, (b) MG2, (c) MG3, (d) MG4 and (e) MG5.

**Figure 3:**
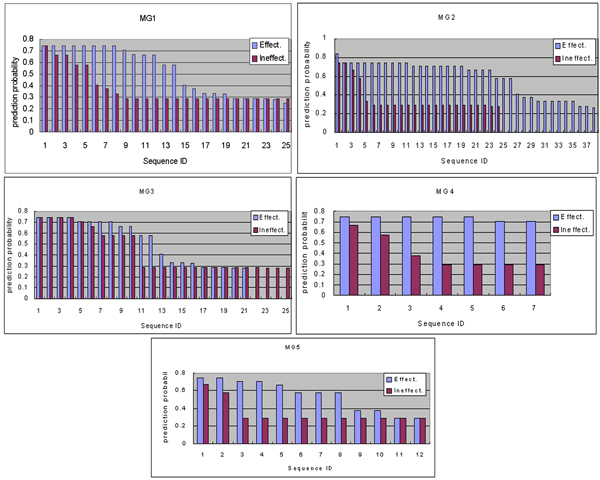
Prediction probability distributions of siRNA sequences effective and ineffective for MG1 to MG5 by the proposed decision tree method. The probabilities for effective ("Effect") and ineffective ("Ineffect") siRNAs computed by using the proposed decision tree method are shown for (a) MG1, (b) MG2, (c) MG3, (d) MG4 and (e) MG5.

After carrying out the learnings of the RBF network and decision tree using 860 effective and 860 ineffective sequences, we obtained eight clustered (C1 to C8) listed in Table 1 and the decision tree diagram shown in Figure 1. Then we computed the prediction probabilities of gene silencing for recently reported individual effective and ineffective sequences for MG1 to MG5 (see Methods) by using the proposed methods. The results are respectively shown in Figures 2(a) – 2(e) and Figures 3(a) – 3(e). Since there were ups and downs in the predicted probabilities of individual sequences, we calculated the average for them.

### Prediction analysis by the RBF network

The average prediction probability of gene silencing for the MG1 effective siRNA sequences was 66.3% with the standard deviation 23.2%, whereas the average probability for the ineffective siRNA sequences was 33.6% with the standard deviation 17.2%. As there is a clear difference between the prediction probabilities of the effective and ineffective siRNA sequences, the predicted probabilities correspond to the effectiveness indication of the proposed method. The average prediction probabilities of effective siRNA sequences for MG2, MG3, MG4 and MG5 were respectively 66% (standard deviation: 17.4%), 57.4% (21.9%), 78.3% (16.7%) and 57.9% (16.7%), whereas the average prediction probabilities of the corresponding ineffective siRNA sequences were 25.5% (19.7%), 40.7% (21.4%), 20.7% (6.2%) and 30.1% (15.4%). As there are also clear differences between the averages of the effective and ineffective siRNA sequences for these genes, the individual predicted probabilities indicate the effectiveness of the proposed method.

**Figure 4:**
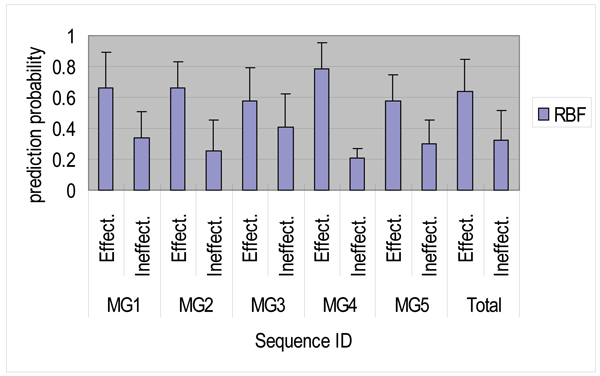
Comparison of the average prediction probabilitiesof the effective ("Effect") and ineffective ("Ineffect") sequences for MG1 to MG5 by the RBF method.

Relations between the average prediction probabilities of the effective and ineffective siRNA sequences for the recently reported siRNAs are shown in Figure 4. With regard to gene classes, MG1, MG2 and MG5 indicate distinctions between the effective and ineffective siRNAs more clearly than MG3 does and MG4 indicates distinctions remarkably clearly. These results therefore imply that there are some differences individual nucleotide frequencies at each position of the siRNAs effective for these gene classes. Although MG3 indicates differences between the effective and ineffective siRNAs, the ratios of the effective to ineffective ones are less than 20%. This implies that there is no big difference between the individual nucleotide frequencies of the siRNAs effective and ineffective for silencing this class of genes. The entire average of 103 effective sequences for these genes was 65% (20.5%), whereas that of 93 ineffective ones was 32% (19.1%).

### Prediction analysis by the decision tree learning

**Figure 5:**
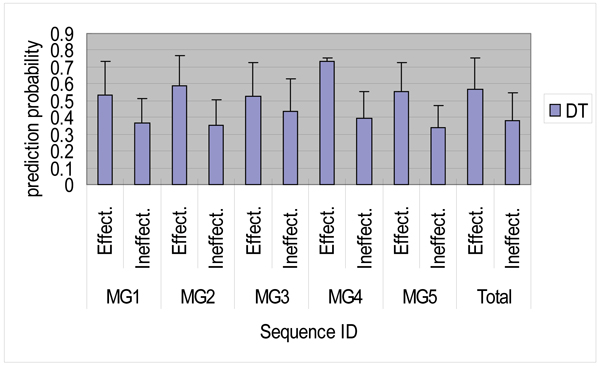
Comparison of the average prediction probabilities of the effective ("Effect") and ineffective ("Ineffect") sequences for MG1 to MG5 by the decision tree learning method.

We also computed the average prediction probabilities for MG1 to MG5 by using the decision tree learning. Relations between the average prediction probabilities of the effective and ineffective siRNA sequences are shown in Figure 5. Comparing Figure 4 with Figure 5, we can understand the differences between the average prediction probabilities of the RBF and decision tree methods. Although the average prediction probability for MG1 effective siRNA sequences was 53% (20%) by the decision tree learning, the corresponding probability by the RBF network was 66.3% (23%). This is 13% higher than that of the decision tree learning. There are similar relations among the average prediction probabilities for MG2 to MG5. The entire average prediction probability of 103 effective siRNA sequences for these genes was 56.6% (18.9%), whereas that of 93 ineffective siRNA sequences was 38.1% (16.3%). Although the method of the RBF network might be superior to that of the decision tree learning, different results imply that two methods have their own prediction criteria.

### Combined method of the RBF network and decision tree learning

**Figure 6:**
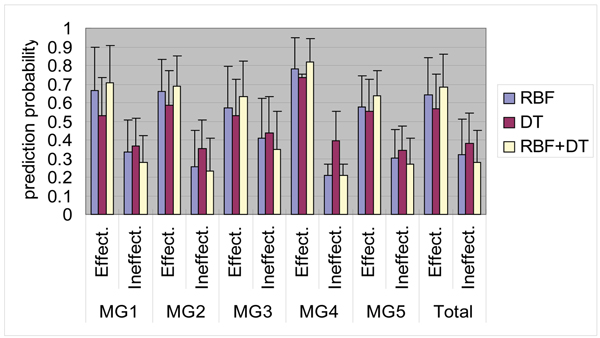
Average prediction probabilities for MG1 to MG5 in this study. Effective ("Effect") and ineffective ("Ineffect") siRNAs are predicted using decision tree learning (DT), RBF network (RBF) and the combined method (DT+RBF).

Since there were different prediction features in the two methods, we combined both methods to increase prediction capability. That is, if a candidate sequence is predicted as a high prediction probability one in either method, it can be inferred as a high prediction probability one. For example, if some sequence in MG2 effective siRNAs were predicted as 50% gene silencing by the RBF network and the same sequence were predicted as 65% one by the decision tree learning, it can be considered as 65% gene silencing in the combined method. The average prediction probabilities of gene silencing for various genes by using the combined method are shown in Figure 6. It is clear that the combined method indicates better prediction probabilities for MG1 to MG5 than those by the RBF network and decision tree learning. The average prediction probabilities for the total effective and ineffective siRNA sequences are respectively 68.5% (17.7%) and 28.1% (17.1%).

### Comparison with other reported methods

The proposed methods use the supervised learning techniques by the RBF network and decision tree for selecting effective siRNA candidates, whereas most of the previous methods use scoring techniques [27]. Although the proposed methods can estimate the probability of gene silencing in the range from 0 to 1, the scoring methods cannot indicate this probability. The scoring method basically sets score values for candidate siRNA sequences according to the designated design rules. Consequently if an siRNA candidate for a specific gene completely satisfies the required design rules, it is expected to get a high score. Even though a high-potential siRNA would be obtained, however, it is difficult to estimate the probability that this siRNA would actually accomplish the expected gene degradation. In addition, as the previous scoring methods are dependent on their designated rules, the obtained scores vary depending on the individual rules. It is therefore quite difficult to compare these different scoring methods with the proposed methods.

**Figure 7:**
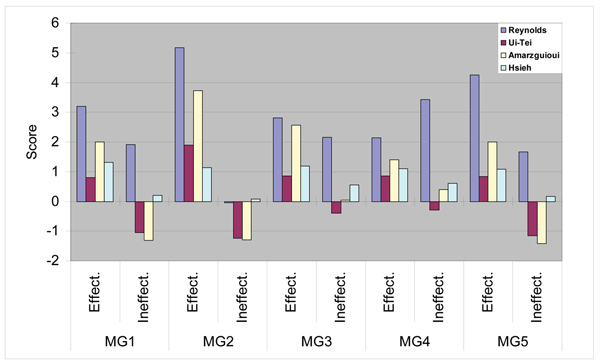
Comparisons to other scoring methods. Scores of the effective and ineffective siRNA sequences were computed on the basis of the positional scores of the individual guidelines shown in Saetrom et al. 2004 [27]. The individual guideline scores are average values of the effective and ineffective siRNAs for MG1 to MG5.

As the important role of the scoring methods is to show the priority of the siRNA candidates, it is necessary to be clear as to score differences between effective and ineffective siRNAs. That is, the scores of the effective siRNAs should be indicated by a set of high values, whereas those of the ineffective ones should be indicated by a set of low or negative values. From this point of view, we examined scores of the siRNAs effective and ineffective for MG1 to MG5 by using the previously reported scoring methods [27]. As a result, it was clear that the previous methods do not always clearly distinguish between effective and ineffective siRNA sequences (Fig. 7). The methods of Reynolds et al. and Hsieh et al., for example, show positive scores for siRNAs effective and ineffective for MG1, MG3, MG4 and MG5 and do not yield distinct differences between the scores of effective and ineffective siRNAs. The average scores of siRNAs for MG4 obtained using the method of Reynolds et al., for example, are in reverse order: That is, the scores of the ineffective siRNAs are larger than those of the effective ones. In addition, although the methods of Ui-Tei et al. and Amarzguioui and Prydz provide correspondences between the individual average scores and the siRNAs effective and ineffective for MG1 to MG5, the relative score differences between the effective and ineffective siRNAs are not large (Fig. 7). In the case of using the method of Ui-Tei et al., for example, the average scores of the siRNAs effective and ineffective for MG1, MG3, and MG4 are respectively 0.8 and -1, 0.86 and -0.4, and 0.86 and 0.29. These results imply that this method might result in producing many same-score siRNA candidates because of the difficulty of setting the candidate priorities.

The proposed method, on the other hand, by estimating the gene silencing probability of the siRNA candidates can, as shown in Figure 6, clearly indicate differences between effective and ineffective siRNAs. This therefore implies that the proposed method can easily be used for selecting high-potential siRNA sequences.

## Conclusion

We proposed two prediction methods for selecting effective siRNA target sequences from many possible candidate sequences by using a radial basis function (RBF) network and decision tree learning. They are quite different from the previous score-based siRNA design techniques and can predict the probability that a candidate siRNA sequence will be effective. The proposed methods were evaluated by applying them to recently reported effective and ineffective siRNA sequences for various genes. In addition, we also proposed the combined method of the RBF network and decision tree learning. As the average prediction probabilities of gene silencing for the effective and ineffective siRNA sequences of the recently reported genes by the proposed three methods were respectively 65% and 32%, 56.6% and 38.1%, and 68.5% and 28.1%, the methods imply high estimation accuracy for selecting candidate siRNA sequences. The evaluation results indicated that the proposed methods would be useful for many other genes. It should therefore be useful for selecting siRNA sequences for mammalian genes.

## Methods

### Supervised learning for effective siRNA classifications by using the RBF network

#### Preparation

**Figure 8:**
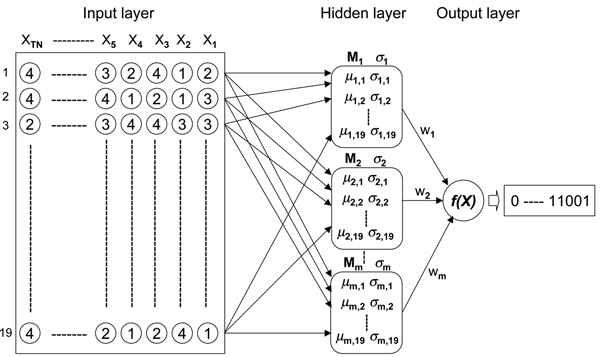
RBF network representation of the relations between effective and ineffective siRNA sequences. The input layer is the set of numerically represented siRNA sequences (A, G, C and T are converted to 1, 2, 3 and 4). Sense strands of siRNAs (cDNAs 5' to 3',19 nucleotides from positions 1 to 19) are described. The hidden layer classifies the input vectors into several clusters depending on the similarities or closeness of individual input vectors. The output layer indicates the effectiveness of individual siRNA sequences (1 : effective gene silencing, 0 : ineffective gene silencing). *X*_*i*_: *i*-th input vector, *TN*: the maximum number of vectors, *M*_*m*_: the location vector, *m*: the number of basis functions, σ: the standard deviation, *w*_*i*_: *i*-th weighting variable, *f(X)*: weighted sum function.

To use a RBF network for selecting effective siRNA sequences, we need to represent individual nucleotides (A, G, C and T) as numerical data. We therefore transform the symbols A, G, C and T into the following numerical representations: A = 1, G = 2, C = 3 and T = 4. Other numerical data representations for individual nucleotides are, of course, also possible. The RBF network can be constructed by adding the hidden and output layers as shown in Figure 8. To carry out the supervised learning for effective siRNA classifications by using the RBF network, we partitioned the data (known effective and ineffective siRNAs) into two sets, one of training data and the other of validation data. The processes of the classifications are carried out two phases: training and validation.

#### Training phase

The training of the RBF network proceeds in two steps. First the hidden layer parameters are determined as a function of the input data (vectors) and then the weights between the hidden and output layers are determined by comparing the target data and the output of the hidden layer. The hidden layer parameters to be determined are the parameters of hyperellipsoids that partition the input data (vectors) into discrete clusters or regions. The parameters locate the center (i.e., the mean) of each ellipsoid's (region or cluster) basis function and describe the extent or spread of the region (i.e., the variance or standard deviation).

The centers of individual clusters are determined as follows:

(1) Randomly choose *m *vectors from the input data set to be the centers of *m *basis functions.

(2) For each vector *i *in the input dataset compute the Euclidean distance *D*_*i*,*m *_to each of the *m *basis function centers.



where *i *is input vector number, e.g., *i *= 1,2, ..., *TN *(the maximum number of vectors in the set of training data, *X*_*i *_is *i*-th input vector, *X*_*i *_= (*x*_*i,1*_, *x*_*i,2*_, ..., *x*_*i,19*_) and *M*_*m *_is the location vector or center of the basis function for hidden node *m*, *M*_*m *_= (μ_*m,1*_, μ_*m,2*_, ...., μ_*m,19*_).

(3) Determine for each input data vector the closest basis function center:

***C***_***bf*,*i ***_= ***Min***{***D***_***i*,1**_**, *D***_***i*,2**_**,...,*D***_***i, m***_}     for *i *= 1,2,...,*TN*,     (2)

where C_*bf*,*i *_is the closest basis function for the input vector *i*.

(4) For all the input vectors grouped around the basis functions, compute the mean C_*m*_



where  is the input vector *i *of the closest basis function *m *and *N*_*m *_is the number of input vectors grouped around the basis function *m*.

(5) Use these grounded means as the new mean values for the *m *basis functions.

(6) Repeat this process until there is no further significant change to the basis function centers.

The number *m *of basis functions starts as a small value – e.g., *m *= 4 – and increases as the validation data is being evaluated. The variances of the individual basis functions (σ_1_, σ_2_, ...,σ_*l*_) are computed after the individual basis functions are determined.

The radial basis function *GR*(*i, m*) for the hidden unit *m *output of the input vector *i *is defined as a Gaussian function in the following way:



where σ_*m*_^2 ^is a measure of the size of the cluster *m *(i.e., the variance or the square of the standard deviation).

Then all that remains is to find the linear combination of weights that produces the desired output (target) values for each input vector. Since this is a linear problem, convergence is guaranteed and computation proceeds rapidly. This task can be accomplished with an iterative technique based on the perceptron training rule or with various other numerical techniques. Technically, the problem is a matrix inversion problem:

*T *= *BW*,     (5)

where *T *is the target vector, *W *is the to-be-determined weighting vector and *B *is the matrix of output values from each hidden unit in response to the input data (calculated from the basis functions, e.g., equation (4)). The matrix is usually not square, so a pseudo inverse may be used to give a minimum least-squares solution.

In the case of the supervised learning, we have already obtained gene silencing results for all input vectors, e.g., *i *= *TN*.









Therefore, *w*_1_, *w*_2_, ..., *w*_*m *_are determined by solving the above linear equations.

After determining the weighting variables, we can compute the percentages of effective and ineffective siRNAs in the individual clusters.

#### Validation phase

To evaluate whether the RBF network carried out appropriate (not overtraining) classifications, we verified individual clusters in the classifications by using the validation data. The differences between the percentages of effective and ineffective siRNAs for the training and validation data are computed for individual clusters. If there are few differences between the percentages of effective and ineffective siRNAs for the training and validation data in some classification, we can infer that the classification was carried out appropriately. If, on the other hand, there are large differences between them, we must conclude that the classification was not appropriate. The differences therefore indicate the effectiveness of individual classifications by the RBF network. The summation of the differences – the entire error of this partition (cluster) number *m *– is used to compare the error of this partition with other errors of other partitions (clusters).

#### Determination of the number *m *of clusters

The number *m *of basis functions corresponds to the number of partitions (clusters) and is determined on the basis of the minimum error of the individual clusters by using the validation data. That is, after carrying out several classifications while changing the number *m *of clusters, the errors of individual clusters are checked and the number of clusters yielding the minimum error is the desired number, i.e., the optimal classification.

### Decision tree learning

#### Preparation

#### Attributes or features

Size: 19 nucleotide sequence

Nucleotides: A, G, C and T

#### Training instances

Effective siRNAs: 860 sequences

Ineffective siRNAs: 860 sequences

To carry out the supervised learning for effective siRNA classifications by using the decision tree learning, we partitioned the training instances into two sets, one for the growth of the decision tree (training data) and other for the decision tree pruning (validation data). The processes of the classifications are carried out in two phases: the growth and pruning of the decision tree.

#### The growth of the decision tree

The algorithm, in outline, is as follows:

(1) if all the instances belong to a single class, there is nothing to do (except create a leaf node labeled with the name of that class).

(2) otherwise, for each attribute that has not already been used, calculate the information gain that would be obtained by using that attribute on the particular set of instances classified to this branch node.

The information gain can be computed in the following way (28).



where

*p *is a number of effective siRNA sequences for this attribute and *n *is a number of ineffective siRNA sequences.

The entropy *E(L) *associated with the position *L *is :



where

*v *is a kind of nucleotides, i.e., *i *= 1 = A, 2 = G, 3 = C and 4 = T, and *L *is the sequence position, i.e., *L *= 1, 2, ...., 19.

The information gain is therefore obtained as follows:

*gain*(*L*) = 1(*p*, *n*) - *E*(*L*)     (9)

(3) use the attribute (position) with the greatest information gain as a branch node.

(4) if the information gain becomes less than the specified criterion, stop the growth of the decision tree and create leaf nodes.

#### Decision tree pruning

Working backwards from the bottom of the tree, the subtree starting at each nonterminal node is examined. If the error (misclassification) rate on the validation data improves by pruning it, the subtree is removed. The process continues until no improvement can be made by pruning a subtree.

### Training, validation and evaluation data of the proposed methods

#### Training and validation data

As effective data, we collected 860 effective siRNA sequences (more than 80% gene silencing at the protein level) from 503 different cDNAs reported in references in the PubMed database. We also randomly generated 860 ineffective siRNA sequences as ineffective data. This is because we know that the randomly generated siRNA sequences were less effective in gene silencing as empirical knowledge. These effective and ineffective siRNAs were used as the training and validation data while partitioning the entire data set into various ratios of training data to validation data: 2 to 1, 3 to 1 and 10 to 1. We used 2 to 1.

#### Data used to evaluate the proposed methods

The proposed method was evaluated by using recently reported effective and ineffective siRNAs. These siRNAs were not used for 860 effective siRNAs.

Reynolds et al. recently analyzed 90 siRNAs systematically, targeting every other position of 197-base regions of human *cyclophilin B *mRNA (GeneBank accession no. M60875) [21]. For simplicity, human *cyclophilin B *is symbolized throughout the present paper as MG1. From the 90 analyzed siRNA sequences we selected as effective ones a set of 25 sequences for which MG1 target gene expression is less than 10% and selected as ineffective ones a set of 25 sequences for which MG1 target gene expression is greater than 48%.

Ui-Tei et al. reported 38 effective and 24 ineffective sequences for six genes: *firefly luciferase (PRL-TK), vimentin, Oct 4, EGFP, ECFP and DsRed *[22]. For simplicity, in the rest of this paper all six of these genes are symbolized as MG2.

Amarzguioui et al. reported 21 effective and 25 ineffective siRNA sequences for four genes: *hTF *(accession no. M16553),*mTF *(accession no. M26071), *PSK *(accession no. J272212) and *CSK *(accession no. NM_004383) [18]. For simplicity, in the rest of this paper these four genes are symbolized as MG3.

Takasaki et al. reported 7 effective and 7 ineffective siRNA sequences for the homo sapiens *cyclin B1 *gene (accession no. NM_031966) [28]. For simplicity, in the rest of this paper this gene is symbolized as MG4.

Huesken et al. reported 37 siRNAs for TC10 (accession no. BD135193), UBE2I (accession no. NM_003345) and CDC34 (accession no. NM_004359). We selected the top-ranked 12 effective and the worst-ranked 12 ineffective siRNA sequences for these genes. For simplicity, in the rest of this paper these genes are symbolized as MG5 [29].

These test data sets (MG1 to MG5) are available in Additional File 1.

## Authors' contributions

ST carried out the system design for the RBF network and decision tree learning and their applications to siRNA designs. YK performed the data analysis.

AK participated the data analysis.

## Supplementary Material

Additional File 1 Test data sets (MG1 to MG5) of cDNA sequences used in this study. Gene names are provided. The data has also been classified into effective and ineffective siRNA subgroups. Click here for file

## Figures and Tables

**Table 1:** Clusters generated by the RBF network.

Cluster ID	No. of sequences	Percentage of effective siRNAs (%)
C1	134	94
C2	150	70.7
C3	125	70.4
C4	147	61.9
C5	141	43.3
C6	158	32.3
C7	143	27.1
C8	148	8.1
